# Fight against cancer in Portuguese-speaking African countries: echoes from the last cancer meetings

**DOI:** 10.1186/s13027-019-0222-0

**Published:** 2019-02-15

**Authors:** Lúcio Lara Santos, Hirondina Borges Spencer, Fernando Miguel, Satish Tulsidás, Belmira Rodrigues, Lygia Vieira Lopes

**Affiliations:** 1 Head of Experimental Pathology and Therapeutics Research Group, and member of Surgical Oncology Department, Portuguese Institute of Oncology, Porto, Portugal; 2 ONCOCIR- Education and Care in Oncology - Lusophone and Africa, Porto, Portugal; 3 Member of AORTIC Research, Education and Training Committees, Cape Town, South Africa; 4 Medical Oncology Service, Agostinho Neto Hospital, Praia, Cape Verde; 5 Angolan Institute Against Cancer, Luanda, Angola; 6 Medical Oncology Service, Maputo Central Hospital, Maputo, Mozambique; 7 AORTIC Council member, Cape Town, South Africa; 8 Cancer Unit, Sagrada Esperança Clinic, Luanda, Angola

**Keywords:** Portuguese-speaking countries in Africa, Oncology, Cancer care, Education

## Abstract

Portuguese-speaking countries in Africa include Angola, Mozambique, Guinea-Bissau, Cape Verde, São Tomé and Principe. These countries belong to an interstate organization known as PALOP. In June 2018, PALOP organized a cancer meeting in Praia, Cape Verde, entitled ‘Quality in cancer care, optimization of cancer units, cancer education and training.’ This meeting was supported by faculty from the African Organization for Research and Training in Cancer (AORTIC) and was dedicated to the improvement of cancer care in PALOP countries. The burden of non-communicable diseases, which includes cancer, is increasing rapidly in these countries.. During this meeting, a plan was developed to guide the future strategic actions in this community. The main points of action include to increase access to cancer care, boost HPV and hepatitis B vaccination, improve access to cancer treatment, including radiotherapy and palliative care, amongst others. Efforts will be made to ensure the participation of all of these countries at PALOP meetings, including Equatorial Guinea, a potential new member.

## Introduction

**Fig. 1:**
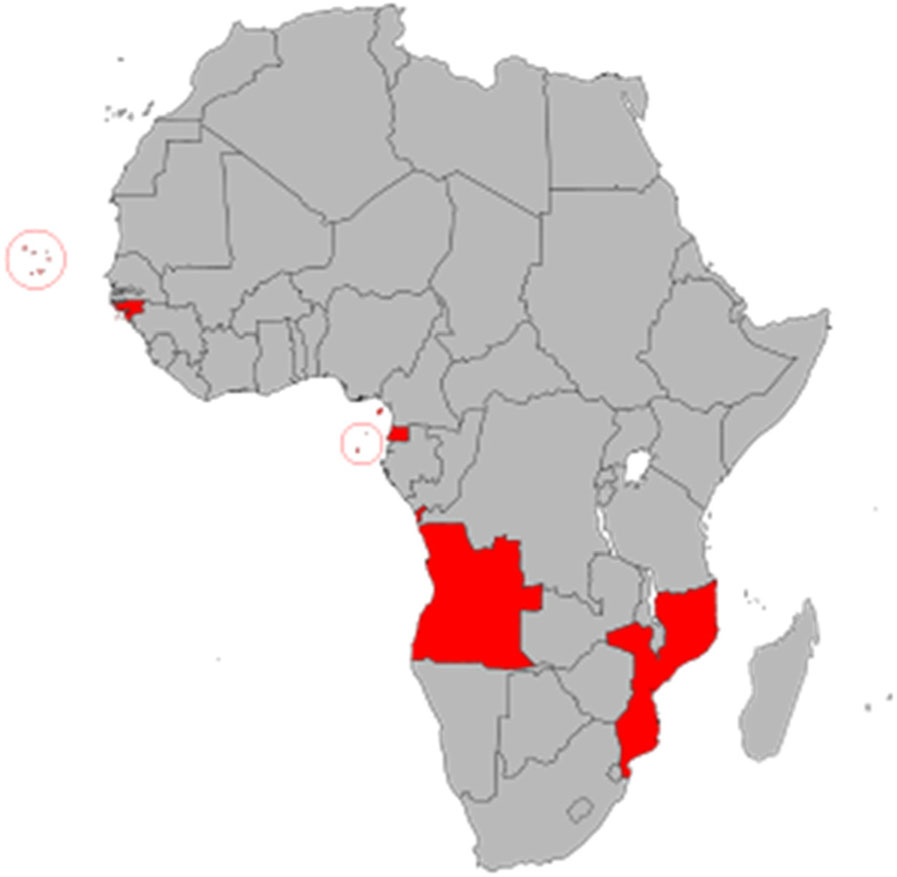
African Countries with Portuguese as the official language

Portuguese-speaking countries in Africa include Angola, Mozambique, Guinea-Bissau, Cape Verde, São Tomé and Principe. In 1992, these countries formed an interstate organization known as PALOP, an acronym that translates into Portuguese-speaking Countries in Africa (‘*Países Africanos de Língua Oficial Portuguesa*’) (Fig. 1) [1]. Apart from having a common language, the PALOP community shares a strong cultural identity, a similar system of governance and a long tradition of contacts and exchanges amongst themselves. These countries also have similarities in their health profile, health resources and experiences and as in most developing countries, a high burden of non-communicable diseases. While infectious diseases continue to pose major challenges, the most significant of these chronic and non-communicable diseases are cardiovascular related diseases, diabetes and cancers [2]. PALOP countries are facing the same challenges in the healthcare system in the establishment of effective and workable cancer control plans. Solutions for the management of cancer needs to be sustainable, local, reality-based and most importantly, supported by government [3]. In order to better coordinate actions to control cancer, the oncologists from PALOP countries that attended the 9th AORTIC International Conference in Durban, South Africa came together and decided to work together. This decision was clearly influenced by the ALIAM (Alliance of African and Mediterranean Francophone Leagues against Cancer), which was created in 2009 with the support of the French League Against Cancer. ALIAM is an advocate in the fight against cancer through education of the population, improvement of patient care, mobilization of the public and private health decision-makers and federate energies and expectations [4].

### Demographics, economic and social data

Demographic, economic and social data statistics are produced with the main goal of providing information about the countries’ structure and its various sectors and advise policy-makers in the decision-making process. However, data published in this area as in others, are often approximations due to the inaccuracy in the resources of these countries is represented in Table 1. Aggregate relevant data from the PALOP region. This data will help to understand the classified profile of this community of countries.

**Table 1:** PALOP, demographics, economic and social data statistics

Country	Population (millions) 2018^5^	2018 Births per 1000^5^	Population Deaths per 1000^5^	Rate of Natural Increase (%)^5^	Infant Mortality Rate^5^	GNI per Capita, PPP 2017^5^	Total Fertility Rate^5^	GDP (US$) 2017 (million)^6^
Angola	30.4	45	10	3.5	44	6060	6.2	124,209.39
Mozambique	30.5	39	10	2.9	65	1200	5.3	12,333.86
Guinea-Bissau	1.9	36	11	2.6	73	1700	4.6	1346.93
Cape Verde	0.6	19	9	2.7	54	6570	2.2	1753.74
São tomé and Príncipe	0.2	33	7	2.6	38	3370	4.4	390.87

*GNI* The gross national income, *PPP* The Purchasing power parity, *GDP* The gross domestic product

5 – data collected from reference [28]; 6 - data collected from reference [29]

PALOP is mostly low or lower-middle income countries with a very young population. Wealth is poorly distributed and there is a high rate of poverty and illiteracy [5]. However, life expectancy at birth according to data from the World Bank is on the rise in all PALOP countries (Angola - 62 years; Mozambique – 58 years; Cape Verde – 73 years; Guinea-Bissau – 57 years and São Tomé and Principe – 67 years) [6].

Increased life expectancy, urbanization and the continued rise of infectious diseases associated with cancer is what is seen and observed in PALOP countries. Cancer is rising in sub-Saharan Africa and the region is predicted to have an increase in burden of cancer greater than 85% by 2030. The same is expected to happen in Lusophone African countries [7].

### Cancer in PALOP countries: The magnitude of the problem

Cancer incidence and mortality statistics for the PALOP region are estimations by GLOBOCAN and based on data collected from neighboring African countries, because there is no reliable data available for these countries. The exception is Mozambique, where incidence estimations reported by GLOBOCAN considered local data collected from the Beira regional cancer registry and most recently the Maputo cancer registry. However, there is no data on cancer mortality. According to GLOBOCAN the estimated number of cases from 2018 to 2040 for all cancers, both sexes, and all ages in PALOP region will increase from 43,376 to 92,900 cases [8].

According to the Institute for Health Metrics and Evaluation, cancer in 2017 was the second cause of death in Cape Verde (17.9%), the third in São Tomé and Principe (12.2%), the sixth in Angola (6.5%) and Mozambique (6.1%) and the seventh in Guinea-Bissau (6.4)% [8]. In all countries, there was an increase in the cancer mortality rate [9].

Published data from Angola is gleaned from the *Instituto Angolano de Controlo do Cancer* (IACC) hospital-based cancer registry. Among the 4791 cancer patients that attended from 2007 to 2011, the most commonly diagnosed cancers were breast (20.5%), cervical (16.5%), and head & neck cancer (10.6%), followed by lymphoma (7.2%), Kaposi sarcoma (6.1%), and prostate cancer (4%). From these, 75.8% were confirmed histologically. A total of 76% of patients were under 60 years old, and 10% were less than 15 years old. From all cancer patients treated at the IACC, 77.3% lived in the Luanda province [10].

The Cancer Registry of the Maputo Central Hospital (MCH) studied 1707 cases, 76.6% of which were confirmed histologically. Prostate cancer, Kaposi sarcoma, and liver cancer were the most frequent in men (ASIR: 24.5, 19.8, and 13.3, respectively). Cervical and breast cancers and Kaposi sarcoma were the most common among women (ASIR: 32.4, 11.8, and 9.5, respectively) [11]. Previously, Lorenzoni C et al. studied a total of 12,674 cases of cancer (56.9% females). In males, the most common cancers were those of the prostate, Kaposi sarcoma (KS) and the liver. In females, the most frequent cancers were of the uterine cervix, the breast and KS [12].

Spencer BH et al. studied 730 cases of cancer from the cancer registry of Agostinho Neto Hospital (ANH), Praia, Cape Verde, and the most frequent malignant tumors were: Breast (27.8%), Cervix (12.6%), Prostate (7.4%), Stomach (6.8%) and Colorectal (6.8%). In this study, the histological confirmation rate was 62.7% [13].

No published data exists for Guinea-Bissau and São Tomé and Principe. Therefore, only estimations are available. Currently, there are hospital-based registries in the PALOP main hospitals. Nevertheless, data quality is poor, so they need to improve their record keeping. It is a matter of urgency to establish the population-based cancer registry in Angola, Cape Verde, Guinea-Bissau and São Tomé and Principe. As previously mentioned, Mozambique has two population-based cancer registers, namely in Beira and Maputo, with technical and scientific support from the African Cancer Registry Network.

### Control and prevention of Cancer programmes in PALOP

The 58th World Health Assembly held in May 2005, identified the following outcome-oriented objectives for cancer control:

- Preventable tumours (such as those of the lung, colon, rectum, skin and liver): to avoid and reduce exposure to risk factors (such as tobacco use, unhealthy diets, harmful use of alcohol, physical inactivity, excess exposure to sunlight, communicable agents, including hepatitis B virus and occupational exposures), thus limiting cancer incidence;
- Cancers amenable to early detection and treatment (such as oral, cervical, breast and prostate cancers): to reduce late presentation and ensure appropriate treatment in order to increase survival rates, reduce mortality and improve quality of life;
- Disseminated cancers that have potential of being cured or the patients’ lives prolonged considerably (such as acute in childhood): to provide appropriate care in order to increase survival, reduce mortality and improve quality of life;
- Advanced cancers: to enhance relief from pain and other symptoms and improve the quality of life of patients and their families.

In response to the WHA resolution, WHO, in 2008, WHO published guidelines for effective cancer prevention control programmes in six modules: Planning; prevention; Early detection; Diagnosis and Treatment; Palliative care and Policy and Advocacy [14].

Angola, Mozambique and Cape Verde have issued guidelines, legislation, and decisions in order to build their Cancer Control program as previously stated, but none of these countries officially have an approved and funded program. The other PALOP countries have no information. The common cancers in these countries are Kaposi’s sarcoma, cervical cancer, breast cancer, prostate, liver, esophagus, stomach, colorectal, bladder, head and neck, leukemia and non-Hodgkins lymphoma. Some of these malignancies may be prevented or detected early, but for the moment this does not happen. Screening for cervical cancer, HPV vaccination, and hepatitis B vaccination should be promoted in PALOP countries. Cape Verde has a cervical cancer screening program.

**Fig. 2:**
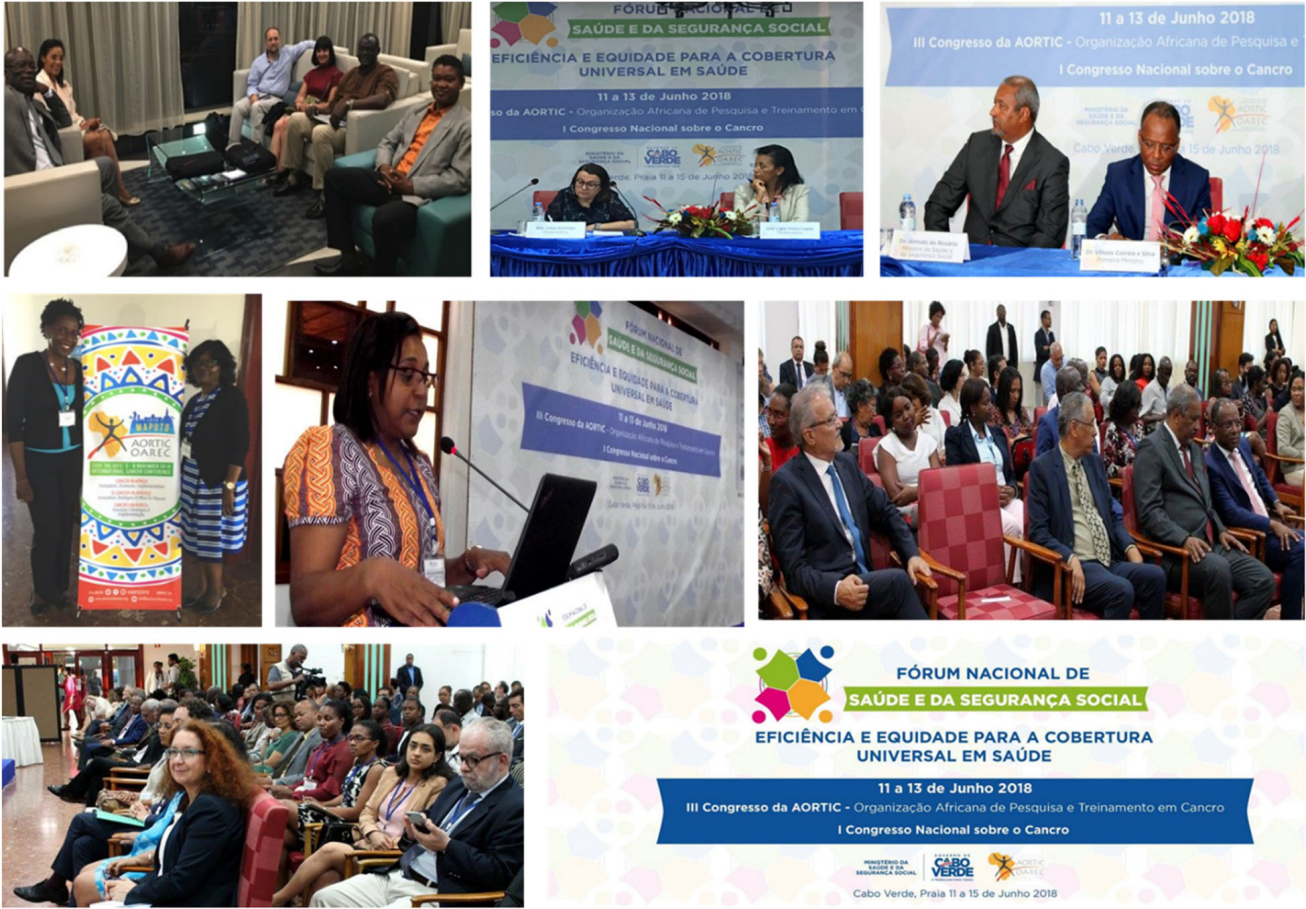
III PALOP cancer meeting and 1st Conference on Cancer in Cape Verde

In order to change this situation, publicize successes and failures and strengthen actions, PALOP oncologists decided to meet every 2 years to evaluate the work done and to organize the following actions, with the scientific support of AORTIC. The first PALOP Cancer Meeting was held in 2014 in Luanda, Angola, the second in 2016 in Maputo, Mozambique, and the third in June 2018 in Praia, Cape Verde (Fig. 2). These meetings concluded that the cancer control activities should be implemented as an integral part of the health delivery system, which must be implemented through a decentralized system where all levels of care will be involved in cancer control.

### Cancer units

The Instituto Angolano de Controlo do Cancer (IACC) in Luanda, Angola, is the oldest public center for the treatment of cancer patients, with radiotherapy, chemotherapy and surgery facilities. Girassol Clinic has radiotherapy and Sagrada Esperança Clinic offers chemotherapy treatment. However, the treatment is not affordable for most patients in Luanda, especially at the Girassol Clinic. In Luanda, units dedicated to cancer treatment are being developed in large hospitals. Also, they are trying to create a multidisciplinary approach in the treatment of this disease and that will act as sources of information for the registry of cancer [15]. The Government announced recently that they will introduce a program to create public oncology units in other provinces of Angola.

**Fig. 3:**
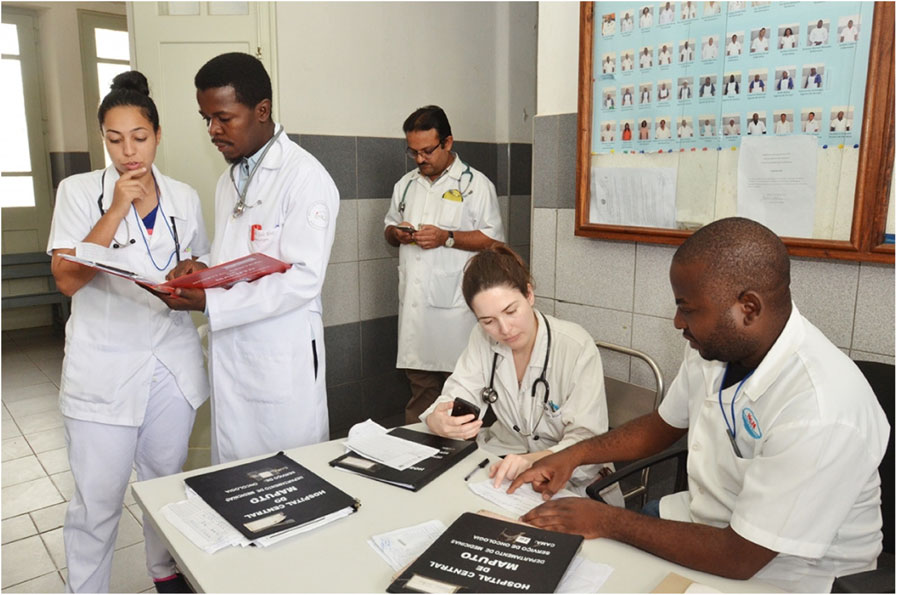
Oncology Unit at Maputo Central Hospital

In Mozambique, dedicated units for the treatment of general cancers and in particular breast cancer have been formed at the Maputo Central Hospital and Nampula Central Hospital. However, it is at Maputo Central Hospital where most cancer cases are treated (Fig. 3) [16]. The radiotherapy unit (with only a linear accelerator) will start operating in 2019.

In Cape Verde, the Agostinho Neto Hospital has a unit for diagnosis and treatment of cancer which includes surgery and chemotherapy and also has equipment for the screening of breast cancer. The majority of patients are transferred to Portugal because there is an agreement between the two countries to carry out radiotherapy or more complex treatments.

In Guinea-Bissau cancer patients are treated mostly at the Simão Mendes hospital and the Catholic Hospital of Cumura [17]. The difficulties at these hospitals are extreme. In São Tomé and Principe, patients are treated in Hospital Ayres de Menezes. In Guinea-Bissau and São Tomé and Principe, only a few doctors are engaged in oncology treatment. Portugal has official cooperation protocols through non-governmental organizations to support the treatment of cancer patients in these two countries.

### Current oncology practice

**Fig. 4:**
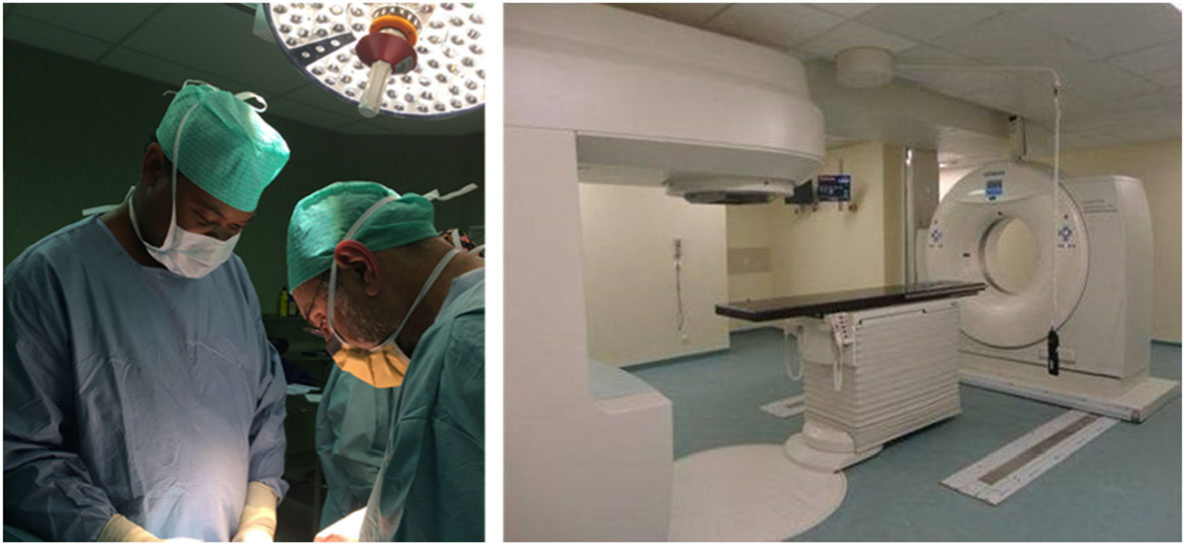
Cancer surgery in Cape Verde and Radiotherapy Unit in Angola

PALOP countries are characterized by an inequitable access to good quality pathology and laboratory medicine services. The lack of quality reagents, adequate pre-analytical procedures, equipment and immunohistochemistry resources are daily difficulties. Another challenge is to properly maintain equipment in working order, replace technical resources and hire skilled human resources. Mozambique has invested heavily in pathological diagnostic resources at Maputo Central Hospital but as in all other countries in this community, they face immense challenges in radiological and endoscopic diagnoses. These difficulties cause delays in diagnosis, staging and initiation of appropriate treatment. According to the PALOP reports, most cancers are diagnosed at advanced stages, which lead to a poor prognosis [18, 19]. Advanced stage at presentation involves complex surgeries, chemotherapy, radiotherapy and palliative care to properly treat these patients. The creation of appropriate facilities and the implementation of cancer education programmes are imperative. The establishment of oncology units and specific resources for the manipulation of hazardous drugs in the region have been the subject of study and sharing of experiences [20, 21]. The option of concentrating cancer experts in one location (IACC - Angola, Maputo Central Hospital - Mozambique and Agostinho Neto Hospital - Cape Verde) allowed gains of efficiency in consultation by specialized tumor boards, improved treatment and training of young specialists that will strengthen the new oncology units (Fig. 4). In the future, these centers of clinical reference and research will carry on clinical trials. The unaffordability of chemotherapy, the adverse side effects and lack of pain medications and quality of cancer surgery needs to be solved. The accessibility to radiotherapy services is an important quality issue of cancer control programmes. According to Wahab M et al. radiotherapy needs is calculated as 64% of incident cases and assuming that one machine treats an average of 450 patients per year [22]. Thus, according to GLOBOCAN 2018 incidence, theoretically, Angola needs 22 machines and there are currently 5 (2 only are in use), Mozambique needs 36 machines and there is currently 1 (is not yet in use), Cape Verde, Guinea- Bissau and São Tomé and Príncipe need a machine but do not have any. Moreover, the existing machines are not always working, making radiotherapy in PALOP countries a problem that clearly needs to be addressed. Angola and Mozambique have invested in the training of its team of radio-oncologists, medical physicists and radiotherapists. Palliative care is still an unmet need in this community of countries.

### Partners on the ground and local community cancer support organizations

The Calouste Gulbenkian Foundation in Portugal developed an oncology patient care program in collaboration with Maputo Central Hospital to strengthen their capacity. This program has now been extended to the Agostinho Neto Hospital in Cape Verde [23]. At the last PALOP cancer meeting, the General Director of Health for Portugal signed an agreement for cancer patients from Cape Verde to be treated in Portugal.

Project ECHO, Mozambique, is a collaborative effort between MD Anderson, three MD Anderson Sister Institutions in Brazil, Maputo Central Hospital (Mozambique) and the Ministry of Health in Mozambique. The Brazilian partners include Barretos Cancer Hospital, Albert Einstein Hospital, and A.C. Camargo Cancer Center [24]. This partnership aims to increase clinical capacity through a comprehensive training program, including regular telementoring, hands-on training workshops, and knowledge-sharing.

The “Health for All Program”, implemented by IMVF (Instituto Marquês Valle Flor) in close partnership with the Ministry of Health and Social Affairs of São Tomé and Principe, is financed by the Camões Portuguese Institute of Cooperation and Language, Fundação Calouste Gulbenkian and the General Director of Health for Portugal [25]. Several non-governmental organizations operate in Guinea-Bissau in the treatment of cancer patients and screening of cervix and uterus in the Cumura hospital [17].

Local community cancer support and non-profit organizations are committed to providing support, advocacy and education and hope for all affected by cancer in the PALOP countries. Examples are the Angolan League Against Cancer and the project “Snowball” dedicated to children in Angola, the Association to Fight Cancer in Mozambique, The Cape Verdean Association for the Fight against Cancer and the Cape Verdean League against Cancer in Cape Verde, the Guinean League against Cancer in Guinea-Bissau and the São Tomé Association of Fight against Breast Cancer and cervix in São Tomé and Principe. These organizations promote annual awareness roadshows and encourage positive action amongst the public, health professionals and policy makers.

### Cancer education programs

Cancer management requires considerable investment in infrastructure, equipment, and personnel. Country cooperation in this regard is critical and is strategic and cost-effective. Training has been one of the most important aspects of the PALOP region as part of cancer control programs in these countries. Existing oncologists were trained locally or in countries such as Brazil, Portugal, Spain and Cuba. The informal training that stems from irregular activities has hampered the consistency and proficient manner to train oncologists in the various areas. In this regard, several interventions to train Mozambique oncologists have been carried out by hospitals in Brazil, the Calouste Gulbenkian Foundation, MD Anderson Cancer Center from USA (Project ECHO) and others [23, 24]. Similar interventions in other PALOP countries have occurred or are planned to be implemented in the next few years, For example, the Central Military Hospital of Angola will start a training program in medical, surgical and gastroenterology oncology in 2019. This is part of the local residency program to strengthen its team of oncologists in Portugal. Therefore, PALOP oncologists consider that formal training and the development of an educational program in oncology should start at university level or at medical schools, during medical residency training and after residency training [26]. The infrastructure for training and accreditation of oncologists is not well established in PALOP countries. A Subspecialty Certificate in Oncology may be the solution to be adopted by the Colleges of Physicians of each country for each medical area responsible for the program, examination and certification. The same should happen with nurses and other health experts involved in cancer care. Another conclusion of this conference was the suggestion of including training programs (including stakeholder programs) under umbrella experts, such as the PALOP School of Oncology. This will allow the program to be evaluated and impact on local professional careers measured. Delegates at the previous PALOP Congress considered that this topic should be addressed at the next AORTIC Conference that will be held 5-8 November 2019 in Maputo, Mozambique during a session on formal cancer education [27]. It was emphasized that the training of mentors and mentees is imperative and AORTIC’s steering committees of education and training should devote themselves to organize a comprehensive training program for oncology specialists from various specialties that will make up the multidisciplinary oncology team.

### Conclusions of the meeting and the plan of action

This conference revealed that Angola has extensive experience in radiotherapy and the handling of hazardous drugs. Mozambique has a population-based cancer registry and pathology and Cape Verde has techniques in the screening of cervical cancer. These competencies are useful for the joint development and upskills of the PALOP Region. During the last PALOP cancer meeting in Cape Verde, the following crucial actions were defined and should be developed, integrated and provided in a timeous manner:

- Cancer awareness and advocacy;
- Population-based cancer registries;
- Improve access to cancer care (globally);
- Screening for cervical cancer and HPV vaccination;
- The hepatitis B vaccine should be boosted;
- Improve clinical evaluation, diagnosis (radiology and pathology) and staging;
- Improve access to cancer treatment (including Radiotherapy);
•Improve the training of nurses, medical oncologists, radiographers and surgical oncologists. The various specialties of the multidisciplinary team also need training;
- The establishment of well-resourced, cancer centres is urgently needed. These centres should be where specialised clinicians, surgeons, pathologists, radiotherapists, nurses, radiologists, pharmacists, and laboratory personnel are given the right conditions to comfortably deliver high-quality care to patients with cancer, at an affordable cost;
- Improve access to palliative care.

Efforts should be made to ensure that Guinea-Bissau, São Tomé and Principe and Equatorial Guinea (a potential new member) participate effectively in PALOP meetings.
